# Non-Data-Aided SNR Estimation for Bandlimited Optical Intensity Channels

**DOI:** 10.3390/s23020802

**Published:** 2023-01-10

**Authors:** Wilfried Gappmair

**Affiliations:** Institute of Communication Networks and Satellite Communications, Graz University of Technology, Inffeldgasse 12, 8010 Graz, Austria; gappmair@tugraz.at

**Keywords:** SNR estimation, optical wireless communications, intensity modulation

## Abstract

Powerful and reliable estimation of transmission parameters is an indispensable task in each receiver unit—not only for radio frequency, but also for optical wireless communication systems. In this context, the signal-to-noise ratio (SNR) plays an eminent role, especially for adaptive scenarios. Assuming a bandlimited optical intensity channel, which requires a unipolar waveform design, an algorithm for SNR estimation is developed in this paper, which requires no knowledge of the transmitted data. This non-data-aided approach benefits to a great extent from the fact that very long observation windows of payload symbols might be used for the estimation process to increase the accuracy of the result; this is in striking contrast to a data-aided approach based on pilot symbols reducing the spectral efficiency of a communication link. Since maximum likelihood, moment-based or decision-directed algorithms are not considered for complexity and performance reasons, an expectation-maximization solution is introduced whose error performance is close to the Cramer-Rao lower bound as the theoretical limit, which has been derived as well.

## 1. Introduction

When comparing radio frequency (RF) to optical wireless communication (OWC) techniques, the advantages of the latter are well known: there are no regulatory and license issues, they are rather inexpensive and easy to deploy, have extremely high throughput and cause no problems with data security, just to mention the most significant aspects in this context [1,2,3,4].

However, not only for RF but also for OWC solutions, the relevant transmission parameters have to be recovered by powerful algorithms, because otherwise subsequent receiver stages, e.g., detectors or error correction algorithms cannot be reliably operated [5,6]. Of course, in case that bandlimited optical intensity solutions are envisaged, a unipolar waveform design is indispensable with respect to pulse shaping and symbol constellation. Investigated in [7,8] for a PAM scheme and root-raised cosines as typical pulse shapes used for RF solutions, this is simply achieved by a suitably selected bias or offset signal. Unfortunately, such concepts are not very efficient in terms of power and energy if no harvesting is implemented. Hence, squared raised cosine and double jump functions have been suggested in [9] as viable alternatives.

Nevertheless, focusing on the pulse shapes proposed in [9] also means that recovery methods developed in the RF context are not automatically applicable. Of course, synchronization of carrier frequency and phase need not be considered in case of intensity modulation, whereas the recovery of the symbol timing is still of paramount importance since this is a pre-requisite for many other estimation and detection procedures. This problem has been tackled in a couple of papers recently published by the author in [10,11,12].

Apart from symbol timing and clock recovery, some knowledge about the signal-to-noise ratio (SNR) is equally important for the reliable transmission of data, e.g., for adaptive communication systems to select modulation and coding schemes according to the given channel conditions so that the link might be operated close to the Shannon bound [13], but also powerful error correction methods—like turbo or LDPC algorithms—need this sort of information [14]. Scanning the open literature, numerous papers are available about SNR estimation in RF channels, e.g., the frequently cited overview by Pauluzzi and Beaulieu [15], but little or no information is published for OWC systems. One of the rare examples is the paper in [16], but the authors discuss on-off keying (OOK), i.e., a binary concept with rectangular pulse shapes and no bandwidth limitation.

This background was the incentive for the author to study in [17] data-aided SNR estimation for a bandlimited optical intensity channel, i.e., data are known to the receiver unit in form of pilot symbols. However, it could be shown that the accuracy of SNR estimates depends on the length of the pilot sequence used for this purpose, although this reduces the spectral efficiency of the communication link as such. Therefore, if SNR estimation could be organized in a non-data-aided (NDA) way by employing payload or user symbols, the error performance of the estimates might be increased by much longer observation windows with no impact on the spectral efficiency. This is the main motivation of the current paper, which is structured as follows:

The signal and channel models used for analytical and simulation work are introduced in Section 2. In Section 3, the Cramer-Rao lower bound (CRLB) is derived as the theoretical limit of the jitter variance for an SNR estimator developed in this respect. Based on the expectation-maximization (EM) principle, an estimator algorithm is introduced in Section 4 and verified in Section 5 by numerical means. Finally, Section 6 concludes the paper.

## 2. Signal and Channel Model

Of course, for the NDA scenario to be investigated in this contribution, we are working with the same signal and channel model used in the companion paper addressing a DA situation [17]. For clarity and readability reasons, this model is briefly recapitulated in the sequel.

Due to the unipolar waveform design mentioned previously, it is assumed that the data symbols *a_k_*, k∈ℤ, are independent and identically distributed (i.i.d.) elements of an *M*-ary PAM alphabet A. In this context, it makes sense to normalize them to unit energy, i.e., $E[a_{k}^{2}]=1$, where E[⋅] denotes the expectation operator. Therefore, by definition of $\eta_{M}=\frac{1}{6}(M-1)(2M-1)$, we obtain $a_{k}\in A=\frac{1}{\sqrt{\eta_{M}}}\{0,\;1,\;\ldots ,\;M-1\}$. As a consequence, the average value is given by

(1) $$\mu_{a}=E[a_{k}]=\frac{1}{\sqrt{\eta_{M}}}\;\frac{M-1}{2}=\sqrt{\frac{3\;(M-1)}{2\;(2M-1)}}.$$

If *h*(*t*) describes the pulse shape satisfying the non-negativity as well as the Nyquist constraint, the signal at the output of the opto-electrical receiver unit can be expressed by

(2) $$r(t)=A\;\sum_{k}a_{k\;}h(t-kT-\tau )+w(t),$$

where *T* is the symbol period and *τ* indicates the propagation delay between transmitter and receiver. The channel gain *A* > 0 is assumed to be a constant, which is justified by the fact that the coherence time of fading events is usually much larger than the observation window needed for estimation purposes. In line with the previous publication about this topic in [17], the noise component in (2) is assumed to be a zero-mean white Gaussian process with variance $\sigma_{w}^{2}$.

In addition, if we introduce the average optical power as $P_{0}=\mu_{a\;}\bar{h}$, where

(3) $$\bar{h}=\frac{1}{\sqrt{T}}\int_{-\infty }^{\infty }h(t)\;dt,$$

the average electrical SNR at the receiver can be defined as

(4) $$\gamma_{s}=\frac{A^{2}P_{0}^{2}}{\sigma_{w}^{2}}.$$

**Figure 1:**
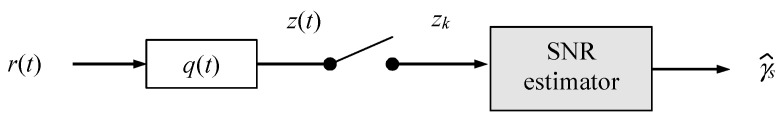
Signal model for SNR estimation.

Nevertheless, before *r*(*t*) is processed in further receiver stages, e.g., in the SNR estimator to be developed in the sequel, it must be filtered appropriately. Assuming an impulse response *q*(*t*), the filter output is determined by z(t)= q(t)⊗r(t), where ⊗ denotes the convolutional operator. For convenient reasons, the signal model used for SNR estimation is illustrated in Figure 1.

It has been proved in [9] that there exists no simple solution for a matched receiver filter in case of a bandlimited optical intensity link. Hence, it is suggested to focus on a solution performing a rectangular shape over the complete spectrum occupied by the user component in (2). By application of the Fourier-transform [18], we have that $Q(f)=ℱ[q(t)]=\sqrt{T}$ for |f| ≤(1+α)/T and Q(f)=0 elsewhere, with *α* as the roll-off factor (excess bandwidth) of the selected pulse shape. In this case, the signal parts of *r*(*t*) and *z*(*t*) are the same, whereas the noise component is determined by *n*(*t*) = *w*(*t*) ⊗ *q*(*t*) representing a zero-mean non-white Gaussian process. Under the assumption that the symbol timing has been reliably recovered and corrected, e.g., by one of the algorithms proposed in [10,11,12], the *T*-spaced samples at the output of the receiver filter are obtained as

(5) $$z_{k}=z(kT)=A\cdot a_{k}+n_{k\;},$$

where $E[n_{k}]=0$, $E[n_{i\;}n_{k}]=2(1+\alpha )\;\sigma_{w}^{2}\;\mathrm{sinc}[2(1+\alpha )(i-k)]$, and sinc(*x*) = sin(πx)/(πx).

## 3. Cramer-Rao Lower Bound

### 3.1. Log-Likelihood Function and Fisher Information Matrix

For parameter estimation, in general, the Cramer-Rao lower bound (CRLB) is a major figure of merit [19]. It turns out to be most helpful for comparison purposes, since the bound represents the theoretical limit of the error performance of any algorithm developed in this context.

By detailed inspection of (4), it is obvious that the average electrical SNR is a function of the channel gain and the standard deviation of the noise component in (2), *A* and *σ_w_*, respectively, whereas *P*_0_ might be considered as a constant factor depending on the PAM scheme and the selected pulse shape. Therefore, it makes sense to focus on the SNR normalized by $P_{0}^{2}$, henceforth denoted by $\rho_{s}=\gamma_{s}/P_{0}^{2}$, and to employ $\rho_{s}$ and $P_{n}=\sigma_{w}^{2}$, instead of *A* and $\sigma_{w}$, as elements constituting the parameter vector **u** to be estimated in the sequel, i.e., $u=(u_{1},u_{2})=(\rho_{s},P_{n})$. On top of that, it is assumed that *L* observables given by (5) are available for the estimation procedure, which might be elegantly expressed in vector form:

(6) z=A⋅a+n.

It is to be recalled that the noise samples in **n** are not independent. The related covariance matrix is given by $E[n\cdot n^{T}]=2(1+\alpha )\;\sigma_{w}^{2}\;\Omega$, where **Ω** describes a symmetric Toeplitz matrix [20] with entries $\omega_{ik}=\mathrm{sinc}[2(1+\alpha )(i-k)]$ for line *i* and column *k*.

Conditioned on the knowledge of the data sequence **a** and the unknown but deterministic parameter vector **u**, the log-likelihood function (LLF) characterizing the estimation problem has been derived in [17] as

(7) $$\Lambda (z|a;u)=-\frac{L}{2}\log P_{n}-\frac{z^{T}\Psi \;z-2\sqrt{\rho_{s}P_{n}}\;z^{T}\Psi \;a+\rho_{s}P_{n}\;a^{T}\Psi \;a}{4(1+\alpha )P_{n}},$$

where $\Psi =\Omega^{-1}$. However, the computation of the CRLB for NDA estimation requires that the LLF does not depend on **a**, which is achieved by averaging the related likelihood function, i.e., $\mathrm{Pr}(z|a;u)=e^{\Lambda (z|a;u)}$, with respect to $a\in A^{L}$, where $A^{L}$ denotes the *L*-dimensional symbol space spanned by *L* i.i.d. elements of A. Therefore, we have that

(8) $$\Lambda (z;u)=\log\mathrm{Pr}(z;u)=\log\left(\frac{1}{M^{L}}\sum_{a\in A^{L}}e^{\Lambda (z|a;u)}\right).$$

As a consequence, the entries of the Fisher information matrix (FIM) are obtained as

(9) $$J_{i,k}=E_{z}\left[\frac{\partial \Lambda (z;u)}{\partial u_{i}}\frac{\partial \Lambda (z;u)}{\partial u_{k}}\right],$$

where $E_{z}[\cdot ]$ denotes expectation with respect to **z**, i.e., an averaging procedure with respect to **a** and **n**, which is only possible by numerical means.

According to theory, the CRLB for parameter *u_i_* is formally given by the *i*-th diagonal entry of the inverted FIM. Since we are only interested in the estimation of *ρ_s_*, representing the first element of **u** in our definition introduced before, the corresponding CRLB develops as

(10) $$\mathrm{CRLB}(\rho_{s})=\frac{J_{22}}{J_{11}J_{22}-J_{12}J_{21}}=\frac{J_{22}}{J_{11}J_{22}-J_{12}^{2}}.$$

### 3.2. Low-Complexity Solution

From the complexity point of view, it is clear that the LLF in (8) is the most demanding ingredient for the computation of the CRLB in (10). This is mainly due to the averaging procedure, which is in the order of $M^{L}$ operations. Even smaller values of *M* and *L* are challenging in this respect, but for values of *L* between 100 and 1000, which are typical for an NDA scenario, the computational load would be intractable. Luckily, for some values of the excess bandwidth, in particular for $\alpha \in \{0,\;\frac{1}{2},\;1\}$, it turns out that **Ω** boils down to an *L*-dimensional identity matrix $I_{L}$, which means that $\Psi \;=\Omega^{-1}\;=I_{L}$. In consequence, the likelihood function in (8) can be re-organized as

(11) $$\mathrm{Pr}(z;u)=\frac{P_{n}^{-L/2}}{M^{L}}\sum_{a\in A^{L}}\exp\left(-\frac{z^{T}z-2\sqrt{\rho_{s}P_{n}}\;z^{T}a+\rho_{s}P_{n}\;a^{T}a}{4(1+\alpha )P_{n}}\right).$$

Because of $\Psi \;=I_{L}$ the elements of **z** might be considered as statistically independent entries. By taking into account that the symbols $a_{i}\in A$ are i.i.d., we just obtain

(12) $$\mathrm{Pr}(z;u)=\frac{P_{n}^{-L/2}}{M^{L}}\sum_{a\in A^{L}}\prod_{k=0}^{L-1}e^{\Lambda (z_{k}|\;a_{i};u)},$$

where

(13) $$\Lambda (z_{k}|a_{i};u)=-\frac{{\left(z_{k}-\sqrt{\rho_{s}P_{n}}\;a_{i}\right)}^{2}}{4(1+\alpha )P_{n}}.$$

Finally, the averaging of Pr(z|a;u) with respect to $a\in A^{L}$ is simply achieved, if we exchange in (12) the order of sum and product, i.e.,

(14) $$\mathrm{Pr}(z;u)=\frac{P_{n}^{-L/2}}{M^{L}}\prod_{k=0}^{L-1}\sum_{a_{i}\in A}e^{\Lambda (z_{k}|a_{i};u)},$$

resulting in a computational complexity in the order of M×L, which is much less compared to $M^{L}$.

In the next step, with Λ(z;u)=logPr(z;u), the first-order derivatives in (9) are expressed by

(15) $$\frac{\partial \Lambda (z;u)}{\partial \rho_{s}}=\sum_{k=0}^{L-1}\frac{\sum_{a_{i}\in A}\Lambda_{s}(z_{k}|a_{i};u)\;e^{\Lambda (z_{k}|a_{i};u)}}{\sum_{a_{i}\in A}e^{\Lambda (z_{k}|a_{i};u)}}$$

and

(16) $$\frac{\partial \Lambda (z;u)}{\partial P_{n}}=-\frac{L}{2P_{n}}+\sum_{k=0}^{L-1}\frac{\sum_{a_{i}\in A}\Lambda_{n}(z_{k}|a_{i};u)\;e^{\Lambda (z_{k}|a_{i};u)}}{\sum_{a_{i}\in A}e^{\Lambda (z_{k}|a_{i};u)}},$$

where

(17) $$\Lambda_{s}(z_{k}|a_{i};u)=\frac{\partial \Lambda (z_{k}|a_{i};u)}{\partial \rho_{s}}=\frac{1}{4(1+\alpha )}\left(\frac{a_{i\;}z_{k}}{\sqrt{\rho_{s\;}P_{n}}}-\;a_{i}^{2}\right)$$

and

(18) $$\Lambda_{n}(z_{k}|a_{i};u)=\frac{\partial \Lambda (z_{k}|a_{i};u)}{\partial P_{n}}=\frac{1}{4(1+\alpha )}\left(\frac{z_{k}^{2}}{P_{n}^{2}}-\sqrt{\frac{\rho_{s}}{P_{n}^{3}}}\;a_{i\;}z_{k}\right).$$

For the simplified scenario, the computation of FIM entries and CRLB does not differ from the general case such that the relationships in (9) and (10) might be used in the same way.

### 3.3. Asymptotic Scenario

For increasing values of *M*, the density of the PAM symbols *a_i_* will increase accordingly, when we assume that their average energy is normalized to unity, i.e., $E[a_{k}^{2}]=1$. Hence, in case that *M* → ∞, the symbol alphabet A turns out to be equally distributed between 0 and $\sqrt{3}$. Applying the framework developed previously to compute the CRLB for such a scenario, it is clear that the sums over $a_{i}\in A$ in (15) and (16) have to be replaced by the related integrals, i.e.,

(19) $$\sum_{a_{i}\in A}a_{i}^{m}\;e^{\Lambda (z_{k}|a_{i};u)}\to {ℐ}_{m}(z_{k};u)=\int_{a_{i}=0}^{\sqrt{3}}a_{i}^{m}e^{\Lambda (z_{k}|a_{i};u)}da_{i},$$

where *m* ∈ {0, 1, 2}. By taking into account the solutions for (19) computed in Appendix A, the first-order derivatives for the FIM entries in (9) are after some lengthy but straightforward manipulations given by

(20) $$\frac{\partial \Lambda (z;u)}{\partial \rho_{s}}=\frac{1}{4(1+\alpha )\sqrt{\rho_{s\;}P_{n}}}\sum_{k=0}^{L-1}\frac{z_{k}{ℐ}_{1}(z_{k};u)-\sqrt{\rho_{s\;}P_{n}}\;{ℐ}_{2}(z_{k};u)}{{ℐ}_{0}(z_{k};u)}$$

and

(21) $$\frac{\partial \Lambda (z;u)}{\partial P_{n}}=-\frac{L}{2P_{n}}+\frac{1}{4(1+\alpha )P_{n}^{2}}\sum_{k=0}^{L-1}z_{k}^{2}-\frac{1}{4(1+\alpha )}\sqrt{\frac{\rho_{s}}{P_{n}^{3}}}\;\sum_{k=0}^{L-1}\;\frac{z_{k}{ℐ}_{1}(z_{k};u)}{{ℐ}_{0}(z_{k};u)}.$$

It is to be recalled that the simplified computation of the CRLB applies in the strict sense only to values of $\alpha \in \{0,\;\frac{1}{2},\;1\}$, where $\Psi =\Omega^{-1}=I_{L}$. However, since the entries of **Ω** are given by $\omega_{ik}=\mathrm{sinc}[2(1+\alpha )(i-k)]$, it is clear that the off-diagonal elements of the matrix are rapidly decaying for $\alpha \notin \{0,\;\frac{1}{2},\;1\}$, which means that **Ω** and **Ψ** might be approximated by an identity matrix of the same dimension such that the simplification would be applicable. This results in a tight approximation of the true bound confirmed by numerical results in Section 5.

## 4. Expectation-Maximization Estimator

Since the normalized SNR value is given by $\rho_{s}=A^{2}/P_{n}$, it is clear that any estimator algorithm must provide the estimates of channel gain as well as noise power, which means that we are focusing in the sequel on a parameter vector **u** = (*A*, *P_n_*). Formally, a maximum likelihood solution for **u** is easily obtained by using the LLF in (8), i.e., $\hat{u}=\arg\max_{\tilde{u}}\Lambda (z;\tilde{u})$. However, this problem cannot be solved in closed form so that we must resort to numerical methods, e.g., the iterative Newton-Raphson procedure [21]. Apart from troubles in terms of initialization, convergence and stability, it is the computational complexity which prohibits this approach, even if the simplified variant with $\Psi =I_{L}$ would be envisaged.

Alternatively, a rather simple solution based on first- and second-order moments given by $E[z_{k}]$ and $E[z_{k}^{2}]$, respectively, delivered reliable estimates only in the very low SNR range. On the other hand, for an algorithm based on symbol decisions [15], useful results were solely achievable for very large SNRs. As a consequence, an expectation-maximization (EM) estimator [22,23,24] is proposed, whose error variance turned out to be close to the CRLB over a wide SNR range as will be demonstrated in Section 5.

The EM algorithm is an iterative procedure using in step *l* the parameter estimates calculated in step *l* – 1. The conditional LLF for this approach is for $\Psi =I_{L}$ expressed by

(22) $$\Lambda (z|{\hat{u}}^{\left(l-1\right)};\tilde{u})=-\frac{L}{2}\log{\tilde{P}}_{n}-\frac{z^{T}z-2\tilde{A}\;z^{T}{\dot{a}}^{\left(l-1\right)}+{\tilde{A}}^{2}{\ddot{a}}^{\left(l-1\right)}}{4(1+\alpha ){\tilde{P}}_{n}}.$$

This is very similar to (7), but **u** is replaced by the trial value $\tilde{u}$ used for optimization purposes; **a** as well as **a***^T^* **a** are substituted by the corresponding soft decisions in step *l* – 1, henceforth denoted by ${\dot{a}}^{\left(l-1\right)}$ and ${\ddot{a}}^{\left(l-1\right)}$. The *k*-th element of vector ${\dot{a}}^{\left(l-1\right)}$ develops as

(23) $${\dot{a}}_{k}^{\left(l-1\right)}=\sum_{a_{i}\in A}a_{i}P_{i,k}^{\left(l-1\right)},$$

whereas ${\ddot{a}}^{\left(l-1\right)}$, representing a scalar, is a finite double sum given by

(24) $${\ddot{a}}^{\left(l-1\right)}=\sum_{k=0}^{L-1}\sum_{a_{i}\in A}a_{i}^{2}P_{i,k}^{\left(l-1\right)}.$$

By inspection of (23) and (24), we observe that the soft decisions are characterized by an averaging of the symbols $a_{i}\in A$ via the *a posteriori* probabilities [25]:

(25) $$P_{i,k}^{\left(l-1\right)}=\mathrm{Pr}(a_{i}\in A|z_{k},{\hat{u}}^{\left(l-1\right)})=\frac{\mathrm{Pr}[z_{k}|a_{i}\in A,{\hat{u}}^{\left(l-1\right)}]\mathrm{Pr}[a_{i}\in A|{\hat{u}}^{\left(l-1\right)}]}{\mathrm{Pr}[z_{k}|{\hat{u}}^{\left(l-1\right)}]}.$$

Because the symbols *a_i_* are i.i.d., we have that $\mathrm{Pr}[a_{i}\in A|{\hat{u}}^{\left(l-1\right)}]=\mathrm{Pr}[a_{i}\in A]=\frac{1}{M}$. Furthermore, by considering the relationship in (13), the probability $\mathrm{Pr}[z_{k}|a_{i}\in A,{\hat{u}}^{\left(l-1\right)}]$ develops as

(26) $$\mathrm{Pr}[z_{k}|a_{i}\in A,{\hat{u}}^{\left(l-1\right)}]=\frac{1}{\sqrt{4\pi (1+\alpha ){\hat{P}}_{n}^{\left(l-1\right)}}}\exp\left(-\frac{{\left(z_{k}-{\hat{A}}^{\left(l-1\right)}a_{i}\right)}^{2}}{4(1+\alpha ){\hat{P}}_{n}^{\left(l-1\right)}}\right).$$

Since the denominator in (25) does not depend on *a_i_*, it might be replaced by a constant including also the *a priori* probability $\mathrm{Pr}[a_{i}\in A|{\hat{u}}^{\left(l-1\right)}]=\frac{1}{M}$, which is determined by the fact that $\sum_{i=0}^{M-1}P_{i,k}^{\left(l-1\right)}=1$.

Finally, computing the first-order derivatives of (22) with respect to $\tilde{A}$ as well as ${\tilde{P}}_{n}$ and equating them to zero for $\tilde{A}={\hat{A}}^{\left(l\right)}$ and ${\tilde{P}}_{n}={\hat{P}}_{n}^{\left(l\right)}$, the parameter estimates for step *l* are achieved in closed form by

(27) $${\hat{A}}^{\left(l\right)}=\frac{z^{T}{\dot{a}}^{\left(l-1\right)}}{{\ddot{a}}^{\left(l-1\right)}}$$

and

(28) $${\hat{P}}_{n}^{\left(l\right)}=\frac{z^{T}z-2{\hat{A}}^{\left(l\right)}\;z^{T}{\dot{a}}^{\left(l-1\right)}+{\left({\hat{A}}^{\left(l\right)}\right)}^{2}{\ddot{a}}^{\left(l-1\right)}}{2L(1+\alpha )}.$$

Nevertheless, the algorithm has to be initialized by appropriately selected values for the probabilities in (25). Since the symbols *a_i_* are assumed to be i.i.d., it makes sense to start the EM algorithm with $P_{i,k}^{\left(0\right)}=\frac{1}{M}$ for all values of *i* and *k*. For convenient reasons, the iterative procedure is summarized as follows:

1. Initialization
  $i=0\ldots M-1,\;k=0\ldots L-1:\;P_{i,k}^{\left(0\right)}=\frac{1}{M}$
2. Iteration: $l=1\ldots l_{\max}$
  - Compute ${\dot{a}}^{\left(l-1\right)},\;{\ddot{a}}^{\left(l-1\right)}$
  - Compute ${\hat{A}}^{\left(l\right)},\;{\hat{P}}_{n}^{\left(l\right)}$
  - Compute $P_{i,k}^{\left(l\right)}$
3. Final estimate
  ${\hat{\rho }}_{s}=\frac{{\left({\hat{A}}^{\left(l_{\max}\right)}\right)}^{2}}{P_{n}^{\left(l_{\max}\right)}}$

It is not difficult to see that the iterative step of the EM algorithm has a computational complexity in the order of *M* × *L* additive and multiplicative operations, only relationship (26) requires the evaluation of square root and exponential functions, which might be elegantly handled via look-up tables. For the numerical results in Section 5, the iterative procedure is organized such that it stops as soon as the relative error between two successive estimates achieves a predefined value of 10^−3^ or when a maximum number of 10^3^ iterations is achieved; by extensive tests it turned out that this would be a good compromise between complexity and accuracy. Furthermore, it is to be remembered that the algorithm applies in the strict sense only to $\Psi =I_{L}$, i.e., $\alpha \in \{0,\;\frac{1}{2},\;1\}$. However, since good results are obtained for other values of *α* as well, it makes sense to employ the EM algorithm in these cases as well.

## 5. Numerical Results

In the following, the EM algorithm developed previously will be verified by numerical means in terms of jitter (error) performance and bias. For comparison purposes, the CRLB is included to the related diagrams, which show also the modified Cramer-Rao lower bound (MCRLB). Derived in closed form in [17], the MCRLB is much simpler than the CRLB, but it is in general less tight [26,27,28,29], i.e., MCRLB(*ρ_s_*) ≤ CRLB(*ρ_s_*), in particular at lower SNRs as demonstrated subsequently.

**Figure 2:**
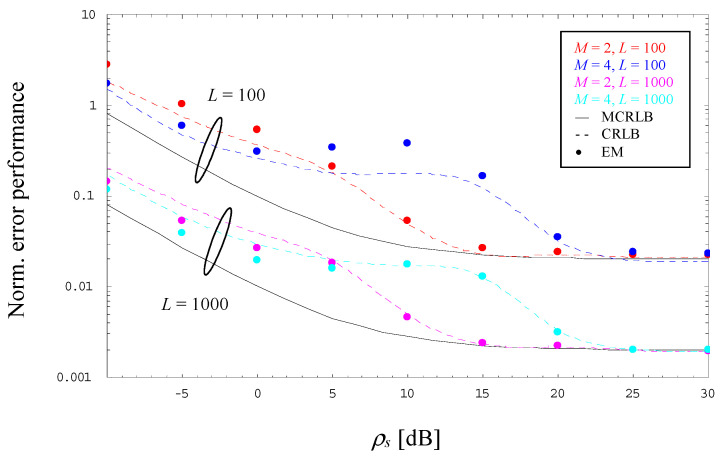
Evolution of the normalized error performance (2/4-PAM, *α* = 0).

For 2-PAM and 4-PAM signals operated with *α* = 0 and two different observation lengths, *L* = 100 and 1000, Figure 2 illustrates the evolution of the error performance as a function of the SNR value; for convenient reasons, error performance and theoretical limits are normalized by $\rho_{s}^{2}$. By detailed inspection, we observe that

**Figure 3:**
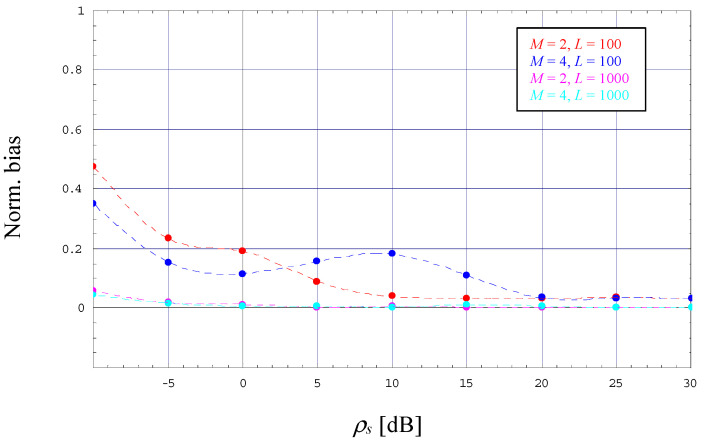
Evolution of the normalized bias (2/4-PAM, *α* = 0).

- For larger SNRs, the CRLB (dashed line) approaches the corresponding MCRLB (solid line) irrespective of the selected modulation scheme and the value of *L*.
- For very low SNRs, the ratio between CRLB and MCRLB seems to approach a small but non-negligible constant, which decreases somewhat by increasing values of *M*.
- In the medium SNR range, we see a significant difference between MCRLB and CRLB whose maximum grows with increasing values of *M* and which moves to larger SNR values.
- For medium-to-low SNRs and *L* = 100, the error performance of the EM estimator, indicated by markers in different style, is characterized by a considerable difference to the CRLB, which shrinks more and more with increasing values of the SNR. This degradation is basically explained by the fact that the algorithm performs a bias effect evolving in the same way, which is depicted in Figure 3 (in this case, the dashed lines do not correspond to an analytical relationship; they are due to an interpolation procedure in order to achieve a better readability of these numerical results). This drawback might be circumvented with larger observation windows, in Figure 2 and Figure 3 exemplified by *L* = 1000.

**Figure 4:**
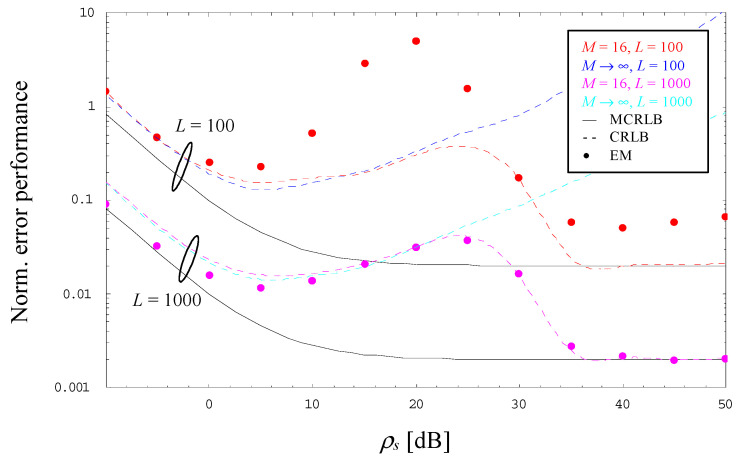
Evolution of the normalized error performance (16-PAM, *α* = 0).

**Figure 5:**
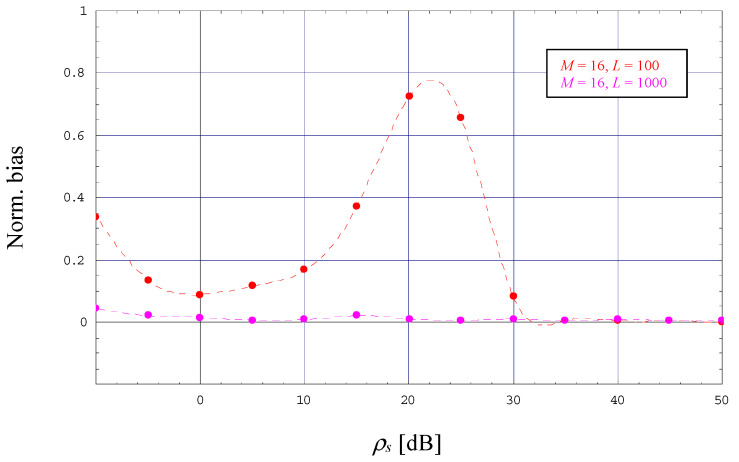
Evolution of the normalized bias (16-PAM, *α* = 0).

The observations made with *M* = 2 and 4 hold also true for higher orders of *M*, in Figure 4 and Figure 5 verified by a 16-PAM scheme operated with *α* = 0 and *L* = 100 or 1000. However, Figure 4 includes also the evolution of the CRLB for *M* → ∞ as it has been derived in Section 4. One can see that the theoretical limit for *M* → ∞ is close to that computed for *M* = 16 as long as the latter does not start to approach the MCRLB, whereas for *M* → ∞ it continues to increase with increasing SNRs. This property suggests that NDA estimation of the SNR becomes more and more problematic in case we increase the order of the selected PAM scheme.

**Figure 6:**
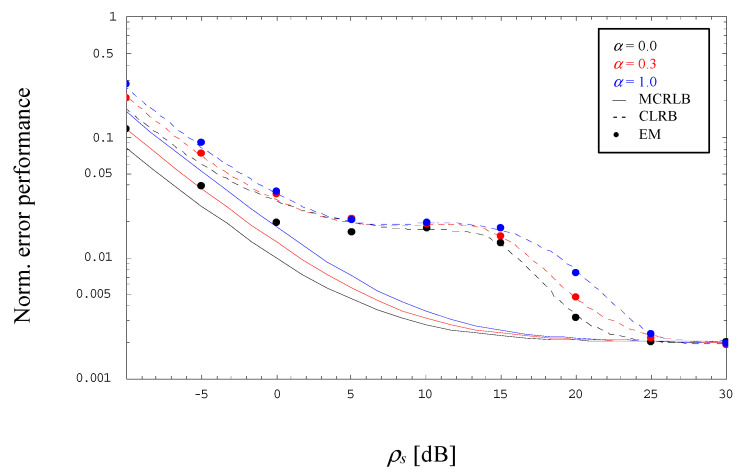
Evolution of the normalized error performance (4-PAM, *L* = 1000).

The diagrams above visualize the error and bias performance for different PAM constellations operated with *α* = 0.0, which is perhaps most interesting in practice, since it represents the scenario with minimum bandwidth. Nevertheless, in order to complete the portrait, Figure 6 shows the normalized error performance for a 4-PAM signal operated with *L* = 1000 and *α* ∈ {0.0, 0.3, 1.0}. According to the exact relationship derived in [17], the MCRLB is proportional to 2(1+α)/L for very low SNRs, whereas for *ρ_s_* → ∞ it approaches 2/L regardless of the selected *α*, or in other words: with respect to *α* = 0, the MCRLB for *α* > 0 appears as shifted to the right depending on the chosen value.

Exactly the same behavior is reflected by the CRLB, although the results are in the very low SNR range somewhat higher than those achieved with the MCRLB, but for rather large values, say *ρ_s_* > 25 dB, the CRLB approaches more and more the horizontal floor characterizing the MCRLB performance. Of course, in the medium SNR range, the CRLB deviates significantly from the MCRLB, which applies also to the simplified computation for *α* = 0.3.

Figure 6 includes also the normalized jitter variance of the EM algorithm developed in the previous section. By detailed inspection, we observe that the performance differs for *α* = 0 in the lower SNR domain somewhat from the CRLB, which is mainly due to a residual bias effect, whereas for medium-to-high SNR values the jitter variances are very close to the corresponding CRLBs regardless of the selected excess bandwidth.

## 6. Concluding Remarks

The availability of reliable SNR estimates is most helpful in many communication systems, particularly when adaptive solutions have to be considered in terms of modulation and coding schemes. This is not only true for radio frequency, but also for optical wireless links. Assuming a bandlimited optical intensity channel, an algorithm for SNR estimation has been developed, which does not require any knowledge about the transmitted data symbols. Such an NDA approach is very appreciated, because the larger observation lengths do not adversely affect the spectral efficiency as it would happen with a DA solution. Maximum likelihood, moment-based and decision-directed methods were out of scope because of complexity and/or performance reasons, but it turned out that the developed expectation-maximization algorithm exhibits an error performance close to the CRLB as the theoretical limit, which is mainly true for longer observation windows where bias effects are negligible.

## Data Availability

Data are available from the author upon mail request.

## Appendix A

In the following, a closed form solution is provided for the integrals specified in (19). Regarding that $\Lambda (z_{k}|a_{i};u)$ is expressed by (13), it is obvious that the integral for *m* = 0 is given by an instance of the error function [30] (3.321/2). Applying the basic rules of integration, we simply obtain

(A1) $$\begin{array}{cc} {ℐ}_{0}(z_{k};u) & =\int_{a_{i}=0}^{\sqrt{3}}e^{\Lambda (z_{k}|a_{i};u)}da_{i}\\ & =\sqrt{\frac{\pi (1+\alpha )}{\rho_{s}}}\;\left[\mathrm{erf}\left(\frac{z_{k}}{2\sqrt{(1+\alpha )P_{n}}}\right)-\mathrm{erf}\left(\frac{z_{k}-\sqrt{3\;\rho_{s}P_{n}}}{2\sqrt{(1+\alpha )P_{n}}}\right)\right]. \end{array}$$

Doing the same for *m* = 1 by taking into account the result in (A1), the corresponding integral is solved as

(A2) $$\begin{array}{cc} {ℐ}_{1}(z_{k};u) & =\int_{a_{i}=0}^{\sqrt{3}}a_{i}\;e^{\Lambda (z_{k}|a_{i};u)}da_{i}\\ & =\frac{z_{k\;}{ℐ}_{0}(z_{k};u)}{\sqrt{\rho_{s}P_{n}}}+\frac{2(1+\alpha )}{\rho_{s}}\left[\exp\left(-\frac{z_{k}^{2}}{4(1+\alpha )P_{n}}\right)-\exp\left(-\frac{\;{\left(z_{k}-\sqrt{3\rho_{s}P_{n}}\right)}^{2}}{4(1+\alpha )P_{n}}\right)\right]. \end{array}$$

Finally, for *m* = 2 and considering (A1) as well as (A2) together with [30] (3.361/1), we get after some algebra

(A3) $$\begin{array}{cc} {ℐ}_{2}(z_{k};u) & =\int_{a_{i}=0}^{\sqrt{3}}a_{i}^{2}e^{\Lambda (z_{k}|a_{i};u)}da_{i}\\ & =\frac{\left[z_{k}^{2}+2(1+\alpha )P_{n}]\;{ℐ}_{0}(z_{k};u\right)}{\rho_{s}P_{n}}\\ & -\frac{2(1+\alpha )}{\rho_{s}^{2}P_{n}}[(\sqrt{3}\rho_{s}P_{n}+\sqrt{\rho_{s}P_{n}}z_{k})\exp\left(-\frac{\;{\left(z_{k}-\sqrt{3\rho_{s}P_{n}}\right)}^{2}}{4(1+\alpha )P_{n}}\right)\\ & \;-\sqrt{\rho_{s}P_{n}}\;z_{k}\exp\left(-\frac{z_{k}^{2}}{4(1+\alpha )P_{n}}\right)]. \end{array}$$

## References

[1] Hranilovic S. (2004). Wireless Optical Communication Systems.

[2] Arnon S., Barry J., Karagiannidis G., Schober R., Uysal M. (2012). Advanced Optical Wireless Communication Systems.

[3] Khalighi M.A., Uysal M. (2014). Survey on free space optical communication: A communication theory perspective. IEEE Commun. Surv. Tutor..

[4] Ghassemlooy Z., Arnon S., Uysal M., Xu Z., Cheng J. (2015). Emerging optical wireless communications—Advances and challenges. IEEE J. Select. Areas Commun..

[5] Mengali U., D’Andrea A.N. (1997). Synchronization Techniques for Digital Receivers.

[6] Meyr H., Moeneclaey M., Fechtel S.A. (1998). Digital Communication Receivers: Synchronization, Channel Estimation, and Signal Processing.

[7] Tavan M., Agrell E., Karout J. (2012). Bandlimited intensity modulation. IEEE Trans. Commun..

[8] Czegledi C., Khanzadi M.R., Agrell E. (2014). Bandlimited power-efficient signaling and pulse design for intensity modulation. IEEE Trans. Commun..

[9] Hranilovic S. (2007). Minimum-bandwidth optical intensity Nyquist pulses. IEEE Trans. Commun..

[10] Gappmair W. (2019). On parameter estimation for bandlimited optical intensity channels. Computation.

[11] Gappmair W., Nistazakis H.E. Blind symbol timing estimation for bandlimited optical intensity channels. Proceedings of the 12th IEEE/IET International Symposium on Communication Systems, Networks and Digital Signal Processing (CSNDSP).

[12] Gappmair W., Schlemmer H. Feedback solution for symbol timing recovery in bandlimited optical intensity channels. Proceedings of the IEEE 4th International Conference Broadband Communications for Next Generation Networks and Multimedia Applications (CoBCom).

[13] Chung T.S., Goldsmith A.J. (2001). Degrees of freedom in adaptive modulation: A unified view. IEEE Trans. Commun..

[14] Summers T.A., Wilson S.G. (1998). SNR mismatch and online estimation in turbo decoding. IEEE Trans. Commun..

[15] Pauluzzi D.R., Beaulieu N.C. (2000). A comparison of SNR estimation techniques for the AWGN channel. IEEE Trans. Commun..

[16] D’Amico A.A., Colavolpe G., Foggi T., Morelli M. (2022). Timing synchronization and channel estimation in free-space optical OOK communication systems. IEEE Trans. Commun..

[17] Gappmair W. (2022). Data-aided SNR estimation for bandlimited optical intensity channels. Sensors.

[18] Proakis J.G., Manolakis D.G. (1996). Digital Signal Processing: Principles, Algorithms, and Applications.

[19] Kay S.M. (1993). Fundamentals of Statistical Signal Processing: Estimation Theory.

[20] Gray R.M. (2006). Toeplitz and Circulant Matrices: A Review.

[21] Press W.H., Teukolsky S.A., Vetterling W.T., Flannery B.P. (1992). Numerical Recipes in C: The Art of Scientific Computing.

[22] Dempster A.P., Laird N.M., Rubin D.B. (1977). Maximum likelihood from incomplete data via the EM algorithm. J. Roy. Stat. Soc. B.

[23] Moon T.K. (1996). The expectation-maximization algorithm. IEEE Signal Process. Mag..

[24] Gappmair W., Lopez-Valcarce R., Mosquera C. (2009). Cramer-Rao lower bound and EM algorithm for envelope-based SNR estimation of nonconstant modulus constellations. IEEE Trans. Commun..

[25] Papoulis A. (1991). Probability, Random Variables, and Stochastic Processes.

[26] D’Andrea A.N., Mengali U., Reggiannini R. (1994). The modified Cramer-Rao bound and its application to synchronization problems. IEEE Trans. Commun..

[27] Gini F., Reggiannini R., Mengali U. (1998). The modified Cramer-Rao bound in vector parameter estimation. IEEE Trans. Commun..

[28] Moeneclaey M. (1998). On the true and the modified Cramer-Rao bounds for the estimation of a scalar parameter in the presence of nuisance parameters. IEEE Trans. Commun..

[29] Gappmair W. (2008). Cramer-Rao lower bound for non-data-aided SNR estimation of linear modulation schemes. IEEE Trans. Commun..

[30] Gradshteyn I.S., Ryzhik I.M. (1994). Table of Integrals, Series, and Products.

